# Anaesthetic Management of a Patient with Thyrotoxicosis for Nonthyroid Surgery with Peripheral Nerve Blockade

**DOI:** 10.1155/2016/9824762

**Published:** 2016-01-17

**Authors:** Mehmet I. Buget, Bilge Sencan, Giray Varansu, Suleyman Kucukay

**Affiliations:** Department of Anaesthesiology, Istanbul University, Istanbul Medical Faculty, 34093 Istanbul, Turkey

## Abstract

Thyrotoxicosis is a hypermetabolic condition caused by an elevation in thyroid hormone levels. The disorder has a variety of causes, manifestations, and therapies. Several clinical features of thyrotoxicosis are due to sympathetic stimulation with increased beta-adrenoreceptor upregulation and sensitization to catecholamine. Anaesthetic management of thyrotoxicosis patients using neuraxial block has been described in literature; however, to our knowledge, there are no reports of peripheral nerve block utilization. Here, we report on the anaesthetic management of a patient with thyroiditis-associated thyrotoxicosis undergoing emergency surgery via a femoral and sciatic nerve block.

## 1. Introduction

The term thyrotoxicosis refers to a clinical state resulting from excessive thyroid hormone action in tissues that is generally caused by elevated thyroid hormone levels [1]. Hyperthyroidism, a form of thyrotoxicosis, is the result of an oversynthesis and oversecretion of thyroid hormones [1]. There are several causes of thyrotoxicosis, including Graves disease, toxic multinodular goitre, toxic adenoma, thyroiditis, and iatrogenic causes. Accurate diagnosis is essential for patients with thyrotoxicosis since its treatment varies according to the cause. For example, in cases of thyrotoxicosis caused by thyroiditis or iatrogenic causes, the administration of antithyroid drugs is contraindicated because in these cases synthesis of thyroid hormones is not increased [2].

Symptoms of thyrotoxicosis are caused by an excess of beta-adrenergic activity and include agitation, tremor, weight loss, sweating, tachycardia, fever, arrhythmia, and heart failure and can lead to death [3]. During the intraoperative and postoperative periods, the patient's cardiac status should be closely monitored along with the potential development of arrhythmias, cardiac ischemia, and congestive heart failure [4].

Patients with thyrotoxicosis presenting for surgery should ideally be made biochemically and clinically euthyroid before surgery, in order to reduce the risk of perioperative thyroid storm. The risk of perioperative thyroid storm is usually higher following an acute event such as surgery, trauma, or infection [3]. Patients with hyperthyroidism who are not adequately clinically prepared for surgery are at serious risk [3]. In the literature there are many of thyrotoxicosis cases managed with general anaesthesia. A limited number of cases preferred neuraxial blockade. However, to our knowledge, this is the first case using peripheral blockade for thyrotoxicosis patient.

In this report, we present a case of perioperative management in a 70-year-old man with uncontrolled thyroiditis-associated thyrotoxicosis, undergoing above knee amputation surgery due to wound necrosis on the tibia.

## 2. Case Report

We report the case of 70-year-old man with peripheral arterial disease, coronary arterial disease (with a history of myocardial infarction and a coronary artery bypass graft procedure that occurred one year previously), and ischemic cerebrovascular disease (with left side hemiparesis that occurred 2 months previously). The patient was admitted to hospital for wound infection. A preoperative electrocardiogram (ECG) revealed sinus tachycardia and a heart rate of 118 beats/min. The results of electrolyte and blood tests were normal with the exception of anaemia (Hb: 9.2 g/dL; Hct: 28.8). The patient did not exhibit thyroid disease or any symptoms of hyperthyroidism before hospital administration and he was not using amiodarone which can be associated with thyroiditis. However, he presented with the following levels: fT4 34.5 pmol/L (normal range is 12 pmol/L–22 pmol/L) and TSH 0.005 mIU/L (normal range is 0.27 mIU/L–4.2 mIU/L). After reviewing the patient's endocrinology, a thyroid scintigraphy was conducted revealing a low uptake, indicating thyroiditis or exogenous iodine intake. Endocrinologists confirmed a diagnosis of thyroiditis-associated thyrotoxicosis and initiation of preoperative propranolol and dexamethasone administration was recommended. After 1 day of propranolol administration, the patient was admitted for surgery. Due to the presence of wound infection, the patient's C-reactive protein levels increased and his general condition became unstable with sepsis diagnosis. Emergency orthopaedic surgery was recommended when the patient's clinical situation worsened.

Upon arrival in the operating room, pulse oximetry and ECG were applied, along with measurements of the patient's invasive blood pressure and temperature. His blood pressure, body temperature, heart rate, and blood oxygen saturation levels were 160/80 mmHg, 36.5°C, 98 beats/min, and 97%, respectively.

We performed an ultrasound-guided femoral and sciatic block with 2% lidocaine and 0.5% bupivacaine. The sciatic nerve block was performed in the lateral position using the Labat technique. A femoral 3-in-1 blockade was conducted with the patient in the supine position. We injected 20 mL of lidocaine and bupivacaine mixture for the sciatic block and 20 mL for the femoral block. We used 40 mL of local anaesthetic mixture which consisted of 15 mL of 2% lidocaine and 25 mL of 5% bupivacaine (patient weight: 85 kg). We checked the efficacy of the blockade before surgery. Presentation of adductor weakness was used to detect the efficacy of obturator blockade. We also administered sedoanalgesia with 2 mg midazolam and 50 *μ*g fentanyl to reduce potential patient stress. A 50 *μ*g/kg/min esmolol infusion was also administered during surgery.

During surgery, the patient's heart rate, systolic artery pressure, diastolic artery pressure, and temperature were in the range of 70–83 beats/min, 130–160 mmHg, 70–85 mmHg, and 35.9°C–36.5°C, respectively.

Due to the potential for cardiopulmonary complications in the postoperative period, intensive care management is essential [2]. Therefore, the patient was transferred to the intensive care unit (ICU) for 48 h close monitoring after surgery. Administration of medication and an esmolol infusion was continued in the ICU. Following this, the patient was discharged from the hospital without serious complication.

## 3. Discussion

Thyrotoxicosis is one of the most common endocrine disorders. Thyroid hormones play an essential role in metabolism. The cardiovascular effects of thyrotoxicosis, including atrial fibrillation, congestive cardiac failure, and ischemic heart disease, are the most important conditions requiring monitoring by the anesthesiologist [5]. Elective surgery and treatment should be postponed until the patient becomes euthyroid. Treatment typically lasts for at least 7 to 10 days, according to the half-life of free T4 [3]. In instances of emergency surgery, such as in the present case, it may not be possible to wait one week for the stabilization of thyroid hormone levels. In this case, we planned to wait for the patient's hormone levels to stabilize before preoperative administration of propranolol was initiated. However, following the admission of the patient to the hospital, his condition worsened due to necrotic wounds. Consequently, emergency amputation surgery was performed.

Thyroiditis is generally associated with thyroid lymphocytic infiltration and positive thyroid antibodies. Antithyroid drugs are ineffective and contraindicated due to the deteriorating effects of thyrotoxicosis [6]. Beta-blocker, steroids, aspirin, and nonsteroidal anti-inflammatory drugs comprise alternative treatment options [6].

For the preoperative management of patients presenting with a thyroid or nonthyroid surgical requirement, the administration of beta-blockers has been shown to be safe and effective [2]. Preoperative propranolol comprised the first-line treatment in the present case. Propranolol is the basic treatment for thyrotoxic patients who are scheduled for surgery [2]. For the prevention of hypertension and thyroid storm, we administered an esmolol infusion in the perioperative period to maintain the heart rate below 90 beats/min. Erturk et al. utilized an esmolol infusion to prevent thyroid storm and maintain haemodynamic stability during a case of mole hydatidiform evacuation complicated by thyrotoxicosis [7]. We also maintained stable haemodynamic parameters during the surgery using an esmolol infusion. Although we did not use magnesium in the perioperative period, it could be beneficial with its well-known sympatholytic effects. During pheochromocytoma surgeries, many authors reported excellent results with magnesium supplement therapy for improving regulation of arterial pressure and heart rate [8].

There are numerous cases of thyrotoxicosis in literature in which surgery was performed under general anaesthesia, while a few cases utilized neuraxial blockade. However, to our knowledge, this is the first case to report utilization of a peripheral blockade. Solak and Aktürk successfully managed a thyrotoxic mole hydatidiform patient under spinal anaesthesia [9]. Varela et al. performed surgery using a combination of general anaesthesia and spinal anaesthesia [10]. We did not just manage a patient with thyrotoxicosis but an elderly patient with limited reserves in the setting of a coronary artery disease and stroke. We did not opt for neuraxial anaesthesia due to the patient's medical history of ischemic cerebrovascular disease. Furthermore, a peripheral nerve blockade can reduce haemodynamic disturbances to a greater extent than both general anaesthesia and neuraxial anaesthesia.

There is the possibility that our case's safety was enhanced by the fact that the patient had thyroiditis rather than hyperthyroidism. Kaderli et al. managed their amiodarone induced thyrotoxicosis patients with general anaesthesia safely while they were in hyperthyroid state in their case series [11]. But our patient's TSH value was much lower than Kaderli et al. cases' mean TSH level and the severity thyrotoxicosis was reported as a significant risk [11].

Our patient may have had more severe thyrotoxicosis than his laboratory values indicated. The normal values for thyroid hormones are determined from patients of all ages and may not apply to sick patients who are 70 years of age or older because current literature supports that serum TSH levels increase with advancing age [12], so that our patient's TSH was effectively lower than suggested and the hyperthyroidism was probably even worse than predicted.

Reports on thyrotoxicosis usually discuss cases of thyroid storm during surgery under general anaesthesia, clinical similarity of thyrotoxicosis with malign hyperthermia, amiodarone-associated thyrotoxicosis, and thyrotoxicosis in mole hydatidiform and thyroid surgery. Case reports on thyrotoxicosis managed under peripheral nerve block are rare. In cases where a thyrotoxicosis patient must undergo emergency surgery and there is no time to administer treatment for making the patient euthyroid, a peripheral nerve blockade and esmolol infusion comprised a suitable alternative method for the anaesthetic management of the patient. For further cases, providing postoperative analgesia via continuous infusion catheter may also be beneficial.

## References

[1] Bahn Chair R. S., Burch H. B., Cooper D. S. (2011). Hyperthyroidism and other causes of thyrotoxicosis: management guidelines of the American Thyroid Association and American Association of Clinical Endocrinologists. *Thyroid*.

[2] Tay S., Khoo E., Tancharoen C., Lee I. (2013). Beta-blockers and the thyrotoxic patient for thyroid and non-thyroid surgery: a clinical review. *Open Access Anaesthetics*.

[3] Farling P. A. (2000). Thyroid disease. *British Journal of Anaesthesia*.

[4] Woeber K. A. (1992). Thyrotoxicosis and the heart. *The New England Journal of Medicine*.

[5] Donangelo I., Braunstein G. D. (2011). Update on subclinical hyperthyroidism. *American Family Physician*.

[6] Franklyn J. A., Boelaert K. (2012). Thyrotoxicosis. *The Lancet*.

[7] Erturk E., Bostan H., Geze S., Saracoglu S., Erciyes N., Eroglu A. (2007). Total intravenous anesthesia for evacuation of a hydatidiform mole and termination of pregnancy in a patient with thyrotoxicosis. *International Journal of Obstetric Anesthesia*.

[8] Dubé L., Granry J.-C. (2003). The therapeutic use of magnesium in anesthesiology, intensive care and emergency medicine: a review. *Canadian Journal of Anesthesia*.

[9] Solak M., Aktürk G. (1993). Spinal anesthesia in a patient with hyperthyroidism due to hydatidiform mole. *Anesthesia & Analgesia*.

[10] Varela A., Yuste A., Villazala R., Garrido J., Lorenzo A., López E. (2005). Spinal anesthesia for emergency abdominal surgery in uncontrolled hyperthyroidism. *Acta Anaesthesiologica Scandinavica*.

[11] Kaderli R. M., Fahrner R., Christ E. R. (2015). Total thyroidectomy for amiodarone-induced thyrotoxicosis in the hyperthyroid state. *Experimental and Clinical Endocrinology & Diabetes*.

[12] Aggarwal N., Razvi S. (2013). Thyroid and aging or the aging thyroid? An evidence-based analysis of the literature. *Journal of Thyroid Research*.

